# On Sclerosis of the Spinal Cord

**Published:** 1885-01

**Authors:** 


**On Sclerosis of the Spinal Cord.** Including Locomotor Ataxia, Spastic Spinal Paralysis and other System-Diseases of the Spinal Cord, their Pathology, Symptoms, Diagnosis and Treatment. By JULIUS ALTHAUS, M. D., M. R. C. P., Senior Physician to the Hospital for Epilepsy and Paralysis, etc., etc. With nine illustrations. New York: G. P. Putnam's Sons, 27 and 29 West Twenty-third street. Price, $2.75. 1885.

The frequency of spinal diseases, both organic and functional, gives the publication of this work of Dr. Althaus great value to the profession. Few have had a larger experience, and the obscurity which surrounds this department of medical science, the author has striven assiduously to brush away, by publishing the results of his observation, accurately taken during twenty-five years of hospital and private practice. Like all works issued by the enterprising publishers, the present one is of a high order of professional merit, and unfolds the clearest exposition of the diseases of which it treats of any we have examined.

